# A Cost-Effective and Efficient Chick Ex-Ovo CAM Assay Protocol to Assess Angiogenesis

**DOI:** 10.3390/mps1020019

**Published:** 2018-05-31

**Authors:** Monali Naik, Pratush Brahma, Manjusha Dixit

**Affiliations:** School of Biological Sciences, National Institute of Science Education and Research, HBNI, PO: Bhimpur-Padanpur, Via: Jatani, Odisha-752050, India; monali.naik@niser.ac.in (M.N.); pratush.b@niser.ac.in (P.B.)

**Keywords:** angiogenesis, cup-CAM, ex-ovo CAM, efficient CAM, cost-effective CAM

## Abstract

The chick chorioallantoic membrane (CAM) is an extra-embryonic membrane, comprised of a high density of blood and lymphatic vessels. CAM has a dense capillary network and is commonly used to study in vivo angiogenesis and anti-angiogenesis in response to potential biomolecules and drugs. Most of the earlier reported CAM assays described the in-ovo method—where the viability of the embryo is higher, but accessibility to the CAM is limited. Ex-ovo CAM methods were previously described that employed shell-less cultures of chick embryos, but the low viability of embryos reduced the overall robustness of the angiogenesis assays. We described a method (named as cup-CAM method) which is more economical, has better accessibility and has significantly improved the viability of the embryo till advanced developmental stages. We could perform this simple yet useful experimentation with the common tools available in the laboratory. We successfully used the cup-CAM method for showing the paracrine effects of conditioned media from tumor cells, on the angiogenesis. This method can be used to assay the angiogenic potential of a drug or protein and to observe the embryonic development of the chick embryo and other related scientific applications.

## 1. Introduction

The phenomenon of the formation of new blood vessels from pre-existing ones, known as angiogenesis, is a crucial process involved in many physiological activities, such as embryonic development, wound healing, inflammation and organogenesis [1]. Tumor growth and metastasis are known to depend on the angiogenesis; thus, targeting angiogenesis could be one of the important strategies to arrest tumor growth and invasion into tissues [2].

Various animal model systems were developed, ranging from zebrafish to mice, to study the process of angiogenesis and to observe the effects of pro-angiogenic and anti-angiogenic factors [3,4,5,6,7]. The chorioallantoic membrane (CAM) assay has become the method of choice [8,9]. The CAM model represents one of the most powerful and popular tools for analyzing the angiogenic potential of purified factors and intact cells. The CAM is formed by the fusion of the mesodermal layers of two developmental structures; the Allantois and the Chorion of the avian embryo. Being an immune-compromised but a highly-vascularized tissue, the CAM serves as an ideal indicator of the anti-angiogenic or pro-angiogenic properties of test compounds [10]. The study of putative angiogenic regulators in the CAM was initially used with in-ovo setups. The setup was ideal—with almost natural embryo survivability rates—but encountered problems while accessing and visualizing the CAM, due to the limited size of the window made on the eggshell for application of test molecules. This scenario posed an important concern as limited access, and visibility of the CAM rendered the angiogenesis experiments meaningless. Hence, other options were explored. Finally, ex-ovo setup came into play as it provided scope for better visual access of the CAM. This imposing advantage of the ex-ovo CAM assay offered a clear stronghold to understand the angiogenesis over the in-ovo CAM assay. This method came with limitations such as very low embryo survivability, compared to the in-ovo model. We made prior attempts to improve the embryo survivability in the ex-ovo model. We carried out various setups using Petri dishes, weighing boats and cylindrical vessels such as glasses/tumblers. However, these methods had a major drawback—attributing to the very low survivability of the embryos outside the eggshell. Even with many trials focused on improving the embryo survivability rates, we achieved only a maximum of 50–60% embryo survivability. In this study, we successfully developed highly efficient and cost-effective ex-ovo setup for the CAM assay using plastic cups (ex-ice cream cups). Herein, we name the improvised method as the cup-CAM assay. This method ensures an embryo survivability of 85–90%, which is close to what we observed with in-ovo conditions. In combination with the cost-effectiveness of the materials involved, the increased accessibility to the CAM improved embryo survivability. The easy analysis renders this method a powerful tool to study the angiogenesis in context of a wide range of biological factors.

## 2. Experimental Design

We explained in detail through this protocol, a cost-effective version of ex-ovo CAM assay—which we carried out employing simple facilities available at a research laboratory—with high efficiency.

The ex-ovo CAM assay exploited the highly vascular chorioallantoic membrane to understand the angiogenic potency of various biomolecules or drugs. The ex-ovo assays were conducted by culturing the egg components, i.e., embryo, yolk and albumin outside the egg shell from the 3rd embryonic day onwards. After culturing the embryo until the 7th embryonic day, a disc soaked with the test component was placed on the region where two major blood vessels bifurcate. Upon impregnation of the disc, the embryo was grown till the 13th embryonic day. On the 13th embryonic day, the images of the region containing the disc on the CAM were taken with the help of a stereomicroscope. The complete CAM image was also taken with the help of a charge-coupled device (CCD) Camera to aid in the final quantification of the angiogenesis. We quantified this assay by manually counting the number of blood vessels arising centrifugally from the discs—conducted by the experimenter and two unbiased observers—independently. We found this process to be a simple method that can be used in future experiments to assay the angiogenic potential of a drug or biomolecule and to observe the embryonic development of the chick embryo and other related scientific applications.

### 2.1. Materials

- 3-day old chicken embryonated eggs (from Central Poultry Development Organization, Bhubaneswar, India).
- Wheat husks, for maintaining temperature and humidity for the egg during transportation.
- Alcohol (Changshu City Hongsheng Fine Chemical Co., Ltd., Changshu City, China).
- An egg-tray, for holding the eggs before culturing in the cups.
- Plastic cups (better if transparent).
- A cuboid metal bar, for cracking the eggshell.
- Scissors, for cutting the eggshell after cracking—to ensure proper transfer of embryonic contents into the cups and for cutting the cling wrap.
- Cling wrap, for covering the shell-less culture.
- Rubber bands, for holding the cling wrap on the plastic cups.
- Toothpicks, for making pores on the cling wrap to provide ventilation for the embryo.
- Whatman filter papers (thickness 1 mm), for making circular discs impregnated over the CAM.
- Forceps, for dropping the discs over the CAM.
- Dulbecco’s Modified Eagle Medium (DMEM; Hi-Media, Mumbai, India, Cat. No.: AL219A).

### 2.2. Equipment

- Cell culture Dishes (60 × 15 mm; Eppendorf, Hamburg, Germany, Cat. No.: 30701119).
- A CO_2_ Incubator (New Brunswick. Galaxy^®^ 170R; Eppendorf).
- A stereomicroscope (Leica–EZ,; Leica Microsystems (UK) Ltd., Milton Keynes, UK).
- A Charge-coupled Device (CCD) Camera (ChemiDoc™ XRS; Bio-Rad, Hercules, CA, USA).

## 3. Procedure

Herein, we describe the detailed procedure to carry out the cup-CAM assay. All the experiments should be done in germ-free conditions of a cell culture facility to maintain sterility. The outer surfaces of the eggs need to be cleaned with distilled water instead of alcohol, as alcohol has been reported to increase the embryonic mortality. The experimental steps need to be carried out in laminar flow hood, thus maintaining sterility. Sanitization of all of the equipment with 70% alcohol prior to the experiments should be done.

### 3.1. Incubation of the Eggs. Time for Completion: 2–3 h

1. 3-day old specific pathogen-free embryonated eggs should be obtained from the hatchery and kept inside the husks during transportation to the lab. (Note: We observed significantly reduced survival rate of embryos when 0-day-old eggs were purchased and stored at lower temperature before proceeding to the experiment.)
2. The presence of an embryo and its location inside the eggs should be checked using an egg candler.
3. The eggs should be placed horizontally in a suitable egg tray and incubated for an hour at 37 °C and 50% humidity without rotation, inside an incubator. (Note: The eggs should be kept horizontally in the incubator for an hour to bring the CAM to the upper side of the egg. This helps in the transfer of an intact and viable embryo to the plastic cups for the ex-ovo culture.)

### 3.2. The Ex-Ovo Culture. Time for Completion: 96–100 h

1. The egg tray containing the eggs should be taken out from the incubator and kept inside a laminar flow hood.
2. The eggs should be kept in a horizontal position and cracked open using the moderately sharp edge of a sterilized hard cube/rectangle shaped structure, lying perpendicular to the horizontal axis of the eggs. (Note: We used a metallic surface for this step to provide a hard and durable material for the cracking of the egg.)
3. The cracked eggs should be placed just above the surface of the cups to avoid any leakage of the egg white. (Note: As the egg white came from the eggs, it is essential that it stays connected to the inner contents of the egg for us to obtain a viable and intact embryo. Additionally, it is essential that the plastic cups used are transparent for better view and should be thoroughly cleaned with 70% ethanol.)
4. The contents of the eggs should be transferred into the cup in a continuous flow by carefully applying the pressure on the crack—without breaking the connection of the inner and the outer contents of the egg or damaging the embryo and the vessels around it. (Note: The contents were efficiently transferred when both halves of the eggshell were separated simultaneously.)
5. The cups should be covered with sterile cling wrap with the help of rubber bands. (Note: Rubber bands were used to ensure that the cling wrap stayed in place, covering the cups.)
6. Small pores should be made on the wrap with the help of sterilized toothpicks to provide ventilation for the chick embryo inside the cup. (Note: Pores should be made carefully to avoid larger pores which could allow the movement of contaminant particles along with air. We ensured that there was no damage to the embryo while making these pores.)
7. The ex-ovo cultures should be kept back in the incubator for the next 96 h and should be maintained at 37 °C and 50% humidity. (Note: We used cell culture incubators for incubating the eggs. We maintained the humidity by keeping a water tray at the bottom of the incubator.)

### 3.3. Preparation of the Conditioned Media. Time for Completion: 120–144 h

1. The tumor cells—of interest from all experimental groups, should be cultured in complete media.
2. To prepare the conditioned media, equal number of cells should be plated in 60 or 100 mm dishes for all experimental groups.
3. Upon reaching 50–70% confluency, the complete medium from the dishes should be replaced with low-serum or serum-free DMEM (Dulbecco’s Modified Eagle’s Medium). (Note: The amount of serum for each cell line needed optimization.)
4. At 24–72 h post incubation, the conditioned media should be collected from the dishes and centrifuged at 4000 rpm for 3 min. (Note: We might have alternatively filtered the conditioned media using a 0.45 µm syringe filter to get rid of cell debris.)
5. The conditioned media can be snap-chilled prior to use for later experiments or lyophilization. (Note: The lyophilized media from each group should be re-suspended in an equal volume of serum-free DMEM and stored in small aliquots for CAM experiments.)

### 3.4. Application of the Substances to Study the Angiogenesis. Time for Completion: 120–124 h

1. Circular discs can be created from filter papers (thickness 1 mm, 6.30 ± 0.04 mm in diameter) using a hole-puncher. The discs should be autoclaved and dried completely before using them as carriers for the test substances to the CAM.
2. These circular discs should be soaked with equal volumes of the conditioned media from all experimental groups used for the angiogenic studies. (Note: The dilutions may vary according to the experiments.)
3. After keeping the ex-ovo culture back for 96 h (from step 7 of Section 3.2), the cultures should be removed from the incubator and placed into the laminar flow hood. The cling wrap should be removed temporarily.
4. The circular discs should be dropped over the CAM with the help of sterilized forceps. (Note: The discs should be carefully dropped over the region where two major blood vessels bifurcated, for a more efficient observation of the effects of the test substances on the angiogenesis.)
5. Images of the embryo along with the discs should be taken for the end-point analysis of the CAM assay.
6. The cultures should be re-covered with the same cling wrap and maintained in the incubator—in conditions mentioned in step 7 of Section 3.2, for the next 120 h.

### 3.5. End-point Imaging and Image Analysis. Time for Completion: 6–8 h

1. The cups containing the embryos should be taken out of the incubator carefully after 120 h of incubation and then placed under a stereomicroscope/CCD (Charge-coupled Device) camera (in-situ imaging).
2. The image of the region surrounding the disc should be captured using a stereomicroscope (8× magnification) and the image of the entire embryo using a CCD digital camera. (Note: The images from the stereomicroscope helped to estimate the microvessel quantity around the implanted disc while the images from the CCD digital camera aided identification of the microvessels arising centrifugally from the disc by portraying the origin of each microvessel.)
3. The images obtained from the stereomicroscope can be processed for background corrections using imaging software (Fiji ImageJ, Aphelion Dev, etc.) and the manual counting of the microvessels arising centrifugally from the disc can be done. (Note: The manual counting of the vessels should be done by two unbiased observers, independent from the experimenter to obtain authenticated data. We decided if substantial differences between the counting of different unbiased observers were found, it was better to exclude that experimental sample from analysis. To have better power of study begin the experimentation with a higher number of replicates (N = 10) in each experimental group.)
4. The number of micro vessels obtained from each group can be compared using the unpaired *t*-test—plotted with the help of GraphPad Prism.

## 4. Expected Results

We performed two types of experiments, one with previously reported ex-ovo and in-ovo methods and another with our improvised cup-CAM method. We kept the growing conditions the same for all the sets and checked the survivability in each experimental group over the whole experimental period. Apart from survivability, we took accessibility and ease of handling into consideration and assessed the overall efficiency of all the setups.

### 4.1. Low Efficacy of Previously Reported Methods

**Figure 1:**
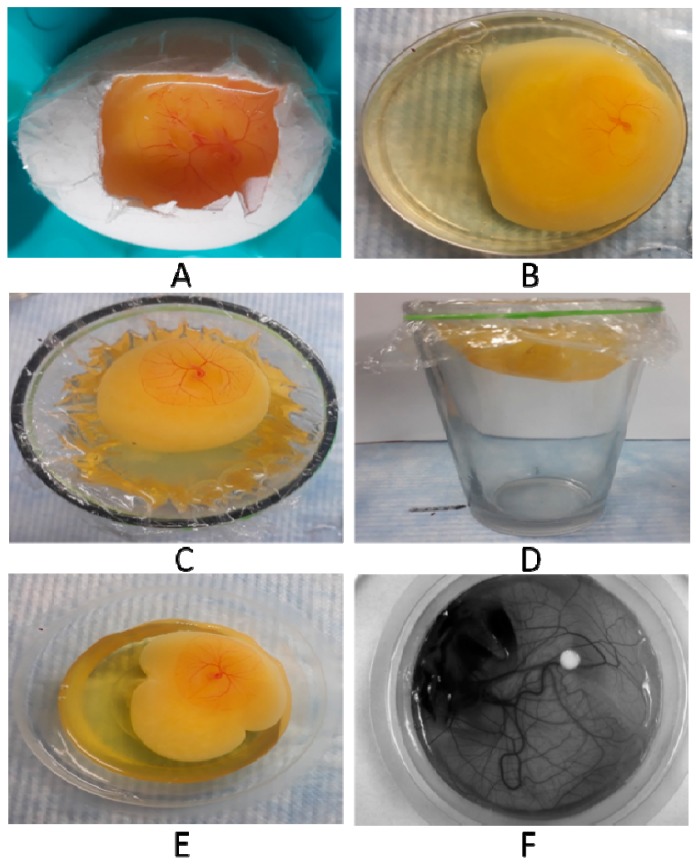
Representative images of chorioallantoic membrane (CAM) variants. (**A**) in-ovo setup by windowing method on day of incubation; (**B**) ex-ovo setup in a Petri plate; (**C**) ex-ovo setup in a glass-vertical view; (**D**) ex-ovo setup in a glass-horizontal view; (**E**) ex-ovo setup on plastic cups, image taken by a Camera and; (**F**) ex-ovo setup in plastic cups, image taken by a Chemidoc (Charge-coupled device (CCD) Camera).

While trying to replicate previously known in-ovo methods (Figure 1A), we realized that the experiment demanded high expertise during handling. Dropping of the CAM by application of pressure pump required high precision—consequently was leading to higher mortality in the in-ovo setup. Inability to accurately drop the CAM and create a window on the eggshell without disrupting the embryonic membranes were found to be the major reasons leading to higher mortality of the embryos. Apart from the survivability issues, there were less accessible surface areas for manipulation during experimentation, and hence the method was not found efficient.

The ex-ovo setups required minimal handling expertise and were easier to replicate. However, in these setups, the embryo mortality (70–80%) arose because of the container used. In the case of Petri dishes (Figure 1B) as the diameter was more, the higher surface tension caused rupturing of the egg yolk—and hence higher mortality. This setup provided higher surface accessibility and required less expertise, but was not a preferred method due to the higher mortality rate. In the case of the glasses (Figure 1C,D) the viability was similar to the cup-CAM method (Figure 1E,F), but due to more depth of the container there was a vigorous movement of the egg content and thus the discs placed were sunken, leading to no favourable outcome. These drawbacks eventually led to overall low efficiency of the previously reported in-ovo as well as ex-ovo methods.

### 4.2. High Efficacy of Improvised Cup-CAM Method

**Figure 2:**
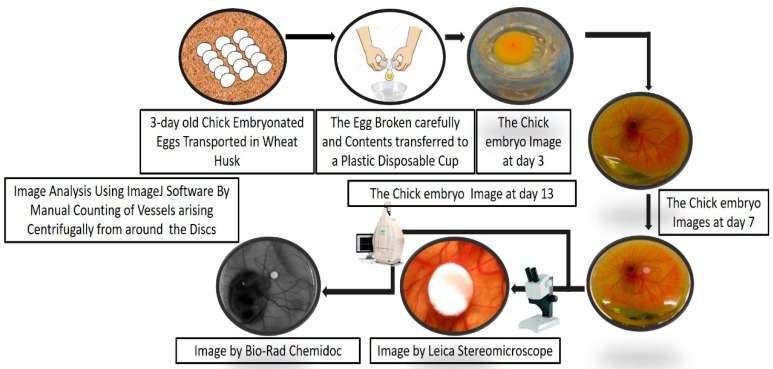
Method overview of the cup–CAM method. After three days from fertilization, incubated chicken eggs were obtained and whole egg content transferred to plastic cups for further incubation. We carried out incubation inoculation of the CAM on the seventh day. On the 13th day, imaging was carried out using a stereomicroscope and a Chemidoc camera.

**Figure 3:**
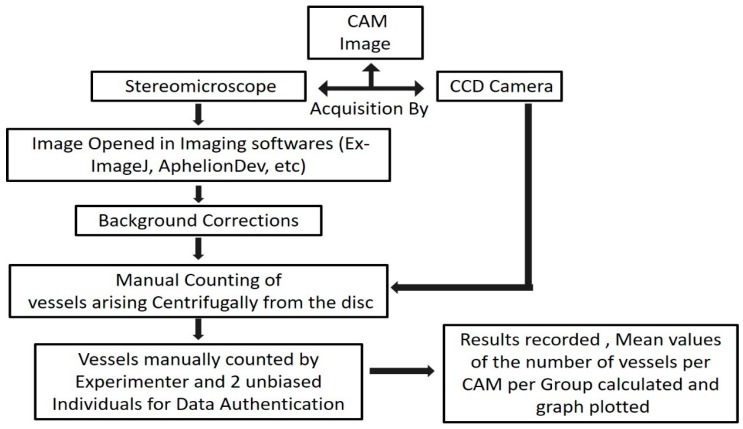
Overview of the CAM assay angiogenesis analysis. The images of the CAM were processed and analyzed by manual counting of the vessels arising from the disc, followed by statistical analysis.

Representative pictures of key steps of the cup-CAM method are shown in Figure 2. Figure 3 shows the important steps in the analysis of the captured CAM images.

**Figure 4:**
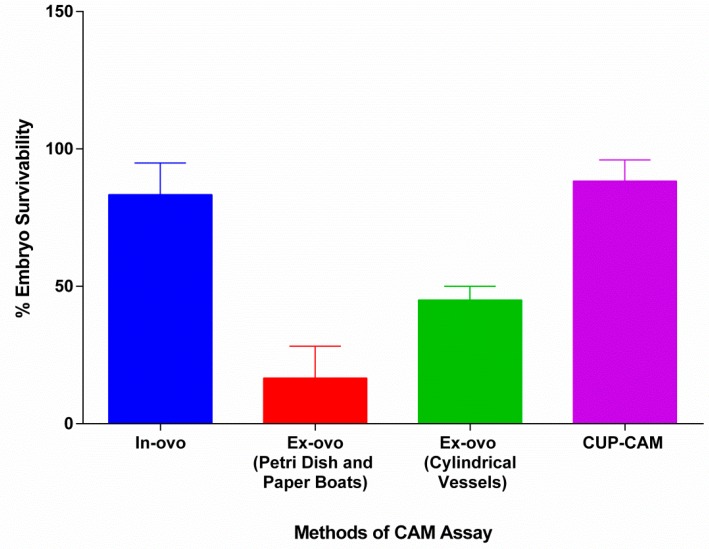
Survivability of various CAM assay techniques. Compared to Petri dishes, weighing paper boats and glasses, ex-ovo cultures in plastic cups gives higher embryo survivability rate. The in-ovo and ex-ovo (plastic cups) show relatively the same survivability.

We found it interesting that we obtained only 5–10% embryo mortality in the ex-ovo method using our preferred approach with the plastic cups. The plastic cups had the right set of dimensions (base diameter = 6–7 cm, mouth diameter = 8–9 cm and height = 3–4 cm) to support proper embryonic growth. The lesser diameter provided appropriate conditions, and hence solved the problem of rupturing due to higher surface tensions. There was also no vigorous movement of the egg content, as was observed in the glasses. Hence the experimental disc placed did not sink nor became displaced in the egg content. Surface accessibility was pretty high and thus was perfect to manipulate various regions of the CAM. By combining all of these factors, this improvised cup-CAM method was found to have higher survivability compared to other methods (Figure 4), full surface accessibility and, to require less expertise. The plastic cups used in this experiment were easily affordable (INR 1 per piece). When these attributes are taken into account to obtain the relative efficiency of different methodologies, the cup-CAM method turns out to be the most efficient (Table 1).

## 5. Discussion

The CAM assay was found to be a very powerful tool to study the angiogenic factors and their role in the angiogenesis. However, the CAM assays require the eggs to grow outside their natural environment, which causes a major restriction. The development of the in-ovo assay was thought to solve this problem. The in-ovo assays presented with other problems. The dropping of the CAM in this protocol by the application of a pressure pump seemed difficult, as it required much expertise and thus led to lower survivability in our experimental setup. The lack of accessibility of the CAM in the in-ovo method added to the lower efficiency of the in-ovo CAM assay.

The lower efficiency of the in-ovo setups and well-known importance of the CAM assay for experimental purposes boosted the development of ex-ovo setups. These setups had high accessibility and easier mode of experimentation, but the embryonic survivability was severely compromised. We found that the lower efficiencies of different ex-ovo setups such as Petri plates is due to the wider base of the plates that results in the spreading of the egg contents—which then leads to rupture and leakage of the yolk. The usage of plastic Petri plates gave rise to an inexpensive ex-ovo CAM assay, but the lower embryo survivability issues demanded the use of a very high number of embryonated eggs. We can attribute lower efficiency of ex-ovo setup in glasses to the movements of the yolk—which due to the narrowness and the depth of the vessel, pushes the testing disks towards the periphery of the vessel. The disks on the periphery can indicate factors about the vessels arising towards the center but fail to provide information on the vessels towards the periphery and hence, make the study meaningless. Our use of the plastic glasses method seemed cost-effective. However, the low viability—as well as the difficulty in analysis due to peripheral movement of the test substance—led to our search for other effective CAM assays.

We found interesting the higher efficiency and viability of ex-ovo setup by growing the chick embryo on plastic cups covered with transparent wraps accompanied by small pores for ventilation. The depth and width of the cups were optimal for gaining higher efficiencies along with slightly modified growth and transport conditions (we used wheat husks for transportation as the husks maintained the natural temperature and humidity required to hatch the eggs). We can attribute the low embryonic death (5–10%) observed in the cup-CAM assay to the deviation from the absolute natural embryonic growth conditions, including required absolute temperature and humidity provided by the mother. As mentioned earlier, the materials used were easily affordable and consequently made it a very feasible CAM assay to study the angiogenesis process.

In conclusion, with the embryonic survivability rate as high as the natural conditions and the ease of experimentation and analysis, this new protocol merges the advantageous qualities of both in-ovo and ex-ovo setups. The method provides an overall highly efficient and cost-effective way to study the phenomenon of the angiogenesis regarding the basic mechanism and drugs for angiogenesis-based therapies.

## 6. Conclusions

We have described a unique and robust method for performing chick ex-ovo CAM assay as a tool for investigating pro and anti-angiogenic agents in vivo. This protocol (cup-CAM), in combination with basic laboratory facilities and low-cost materials, provides the system to conduct a highly efficient angiogenesis assay.

## 7. Reagent Setup

- We thawed DMEM, mixed with amphotericin and filtered through 0.22 um filter and kept the mixture at 4 °C for up to 2 weeks.

## Figures and Tables

**Table 1:** The comparative attributes of various CAM Assay methodologies.

CAM Assay Methods	Attributes			
	No of Eggs Experimented	Accessibility	Embryo Viability	Overall Efficiency
**In-ovo**	30	Low	85–95%	Low *
**Ex-ovo** **(Petri Dishes and Paper Boats)**	15 (Petri Dishes) 15 (Paper Boats)	High	15–25%	Low *
**Ex-ovo** **(Glasses)**	30	High	45–55%	Medium *
**Cup-CAM**	462	High	85–95%	High *

* 1. Low accessibility + 5–95% Embryo viability = Low efficiency; 2. High accessibility + 5–30% Embryo viability = Low efficiency; 3. High accessibility + 40–70% Embryo viability = Medium efficiency; 4. High accessibility + 70–95% Embryo viability = High efficiency; Embryo viability range = 5–95%.
